# My Corporis Fabrica: an ontology-based tool for reasoning and querying on complex anatomical models

**DOI:** 10.1186/2041-1480-5-20

**Published:** 2014-05-06

**Authors:** Olivier Palombi, Federico Ulliana, Valentin Favier, Jean-Claude Léon, Marie-Christine Rousset

**Affiliations:** 1 Department of Anatomy, LADAF, Université Joseph Fourier, Grenoble, France; 2 LJK (CNRS-UJF-INPG-UPMF), INRIA, Université de Grenoble, Grenoble, France; 3 LIG (CNRS-UJF-INPG-UPMF), Université de Grenoble, Grenoble, France

## Abstract

**Background:**

Multiple models of anatomy have been developed independently and for different purposes. In particular, 3D graphical models are specially useful for visualizing the different organs composing the human body, while ontologies such as FMA (Foundational Model of Anatomy) are symbolic models that provide a unified formal description of anatomy. Despite its comprehensive content concerning the anatomical structures, the lack of formal descriptions of anatomical functions in FMA limits its usage in many applications. In addition, the absence of connection between 3D models and anatomical ontologies makes it difficult and time-consuming to set up and access to the anatomical content of complex 3D objects.

**Results:**

First, we provide a new ontology of anatomy called My Corporis Fabrica (MyCF), which conforms to FMA but extends it by making explicit how anatomical structures are composed, how they contribute to functions, and also how they can be related to 3D complex objects. Second, we have equipped MyCF with automatic reasoning capabilities that enable model checking and complex queries answering. We illustrate the added-value of such a declarative approach for interactive simulation and visualization as well as for teaching applications.

**Conclusions:**

The novel vision of ontologies that we have developed in this paper enables a declarative assembly of different models to obtain composed models guaranteed to be anatomically valid while capturing the complexity of human anatomy. The main interest of this approach is its declarativity that makes possible for domain experts to enrich the knowledge base at any moment through simple editors without having to change the algorithmic machinery. This provides MyCF software environment a flexibility to process and add semantics on purpose for various applications that incorporate not only symbolic information but also 3D geometric models representing anatomical entities as well as other symbolic information like the anatomical functions.

## Background

Computer modeling and simulation of the human body is becoming a critical and central tool in medicine but also in many other disciplines, including engineering, education, entertainment. Multiple models have been developed, for applications ranging from medical simulation to video games, through biomechanics, ergonomics, robotics and CAD, to name only a few. However, currently available anatomical models are either limited to very specific areas or too simplistic for most of the applications.

The most generic models used to describe the anatomy are ontologies. Ontologies provide a unified view of a domain of interest resulting of a joint effort of a whole community to standardize a common vocabulary with a clear semantics that can then be shared by users to annotate, index and retrieve data and tools.

A lot of more or less specialized medical ontologies have flourished recently. Most of them are grouped into the Open Biological and Biomedical Ontologies foundry (OBO) [1]. For human anatomy, the reference domain ontology is the Foundational Model of Anatomy (FMA) [2] which is a comprehensive description of the structural organization of the body. Its main component is a taxononomy with more then 83000 classes of anatomical structures from the macromolecular to the macroscopic levels. The FMA symbolically represents the structural organization of the human body.

The complexity of human anatomy can make it difficult for users to comprehend and interact with the anatomical knowledge embedded. This complexity may explain the gap between available anatomical ontologies and potential users [3]. In practice, anatomical concepts are usually used through the scope of other ontologies. For instance in SNOMED CT [4], anatomical concepts are linked to specific diseases or symptoms. In fact, four of the 8 Open Biological and Biomedical Ontologies (OBO) Foundry ontologies and 39 of the site’s other listed ontologies, cover aspects of the representation of anatomical knowledge. Whilst the OBO Foundry has a stated goal of “creating a suite of orthogonal interoperable reference ontologies in the biomedical domain” [5], most of these ontologies have been developed to address a species-specific need articulated by a community working with a particular model organism.

Relations between anatomical structures and their functions appear to be a relevant knowledge. These structural and functional relationships have been explicitly defined at the level of cells in [6] or at the level a whole organism as the drosophila [7]. Also, Uberon [8] takes into account functions in order to query multiple ontologies of species.

Yet, human body modeling relies on morphological components on the one hand and functional and process descriptions on the other hand. This fundamental interaction between structures and functions has been already highlighted by Smith et al. [9,10]. This philosophical approach relies on the idea that the link between anatomy and physiology must be formalized in an new reference ontology. We try to turn this concept into action.

The ICF is an International Classification of Functioning, Disability and Health (ICF) [11] endorsed by the World Health Organization since 2001. However, its analysis reveals that the current version of ICF exhibits non-conformances to many formal ontological principles [12,13]. The need for a formal description of anatomical functions has been outlined in [14], with some guidelines for getting a separate ontology of anatomical functions based on an ontological analysis of functions in general formal ontologies such as GFO [15] or Dolce [16].

Another limitation is that, despite the proliferation of specialized or general ontologies, they are far from being fully exploited, mainly because they are only seen as a standard common structured vocabulary used for annotating and navigating among resources. Yet, they also come with a formal logical semantics that makes them processable by machines through inference algorithms. However, until now, only few existing works in biomedical ontologies (e.g., [17-20]) take advantage of available automatic reasoners. Most of these works rely on ontologies expressed in OWL and use OWL reasoners that are based on Description Logics [21]. OWL is one the standards recommended by the W3C for the Semantic Web, which enables expressing sophisticated ontological constraints (using Description Logics constructors) but with a high computational complexity in the worst case. RDFS is another W3C standard that is broadly used in particular in Linked Data. Whereas OWL is often seen as an extension of RDF and RDFS, this is not exactly the case, mainly because RDF(S) offers interesting non-first-order features which are not present in the Description Logics at the basis of OWL profiles, like the possibility of treating values both as constants and as classes or properties. In the same spirit, the RDF query language SPARQL makes possible to query at the same time the data and the schema and allows that variables stand for classes and properties. This goes beyond the first-order conjonctive queries typically considered in DL-based settings. Recently, RDF-based semantic environments such as Jena (http://jena.apache.org/) or Cwm (http://www.w3.org/2000/10/swap/doc/cwm) have included logical rules to perform inferences on top of RDF datasets. Logical rules and Description Logics are two orthogonal decidable fragments of first-order logics that have been extensively studied in knowledge representation and in deductive databases. The interest of logical rules (a la Datalog) is that they are easy to read and write for practitioners and they have a polynomial data complexity while allowing expressing complex interaction between properties and recursivity.

When application software provides capabilities to display/select 3D graphic entities, its interactive behavior becomes a mandatory feature to manage the graphic entities attached to the digital objects taking part to this application. In such software, selection functions have been under focus to provide users with efficient means to reach the 3D content they are looking for. The most common approaches for multiple object selection include serial selection techniques that require the user to select objects one at a time, e.g. the ubiquitous ctrl + click (or shift + click) approach, and parallel selection techniques such as brushes, lassos, and selection shapes. However, as Lucas et al. [22] point out, each has certain limitations, especially in 3D. For instance, multiple objects may be difficult to distinguish, isolate, or even see due to occlusion, rendering size, environment clutter, and other display factors. Requiring the user to adjust the view can be tedious, cumbersome, and even burdensome, especially when the number of objects to select is high, and may still fail to make certain objects accessible. This is especially true for display scenes with anatomical entities.

Systems commonly address this issue with an indirect selection technique, that is, by allowing the user to specify the desired selection using an alternate representation such as a model tree or component list. Some systems allow selection by common attribute or provide a more general selection query or search [23]. Such indirect selection techniques are useful, but are generally abstract and less intuitive than direct manipulation techniques. They can become cumbersome if the user has to browse a very large amount of entities, which is the case of anatomical entities of the human body.

In the case of man-made objects, recent advances have been made in this field using additional functional information attached to the components of large digital assemblies [24] using an ontology-based approach. This additional information provides the user with efficient means to select/process groups of components that would be otherwise tedious and error prone to identify [25]. Other interactive approaches, like Oh et al. [26] propose group selection with a dynamically computed hierarchy based on the notion of gravitational proximity. Such approaches are less appropriate for rigid and exact specification of selections, particularly when objects or components are frequently or always in contact with or intersecting each other, e.g. when managing sets of anatomical entities. If man-made objects can take advantage of their modeling process to rely on concepts like geometric constraints [27] and simple spatial structures like repetitive placement of objects along lines or circles [28], such structures do not exist for anatomical entities. It is therefore difficult to rely on spatial structures to display/select 3D anatomical entities.

Our approach for supporting efficient navigation and selection of objects in 3D scenes of human body anatomy is to make explicit the anatomic and functional semantics of 3D objects composing a complex 3D scene through a symbolic and formal representation that can be queried on demand.

In this context, our contribution is twofold: 

• First, we address the lack of a formal description of human body functions and we provide a new ontology, called *My Corporis Fabrica* (MyCF), containing the following items: 

– a taxonomy of anatomical functions conform to the ICF terminology;

– a taxonomy of anatomical structures based on FMA;

– and relations between them and with 3D models, that make explicit how anatomical structures are composed, how they contribute to functions, and also how they can be related to 3D complex objects describing patient-specific body parts, declared as instances of appropriate mesh 3D models used for simulation or 3D rendering;

• Second, we equip MyCF with automatic reasoning capabilities that enable model checking and complex queries answering, and we show the added-value of such a declarative approach for interactive simulation and visualization as well as for teaching applications. In particular, we provide new visualization/selection capabilities to manage and browse 3D anatomical entities based on the querying capabilities incorporated in MyCF.

## Results and discussion

MyCF is an ontology-based tool for automatic reasoning and querying on complex anatomical models. The core of MyCF is a comprehensive anatomical ontology, the novelty of which is to make explicit the links between anatomical entities, human body functions, and 3D graphic models of patient-specific body parts. It is equipped with inference-based query answering capabilities that are particularly interesting for different purposes such as: 

• automatic verification of the anatomical validity of 3D models. Indeed, it is important to select the correct set of anatomical entities that contributes to a simulation, e.g. a simulation of movements where the correct bones, muscles, ligaments, …, are required to set up all the 3D and mechanical simulation parameters. These requirements are very close to the selection requirements described in the ‘Background’ section. They can be regarded as equivalent to a selection operator;

• automatic selection and display of anatomical entities within a 3D scene. Anatomical entities can vary largely in size, can be very close to each other or even hidden by other anatomical entities. The use of geometric means to select useful sets of entities is not suited whereas inference-based queries using human body functions can provide much more suited means. Such selection capabilities are particular relevant for diagnosis for instance;

• training students on anatomical entities participating to a certain body function. Here again, this purpose is close to that of selection functions where the connection between function and anatomical entities provides new means to browse and highlight features of anatomical structures accessible in 3D.

The first version of MyCF has been published in 2009 [29]. This version was limited to anatomical entities. The current version of MyCF has been widely improved by adding (*i*) new anatomical entities with more details than FMA for some body parts, (*ii*) almost 4000 human body functions, and (*iii*) the set of classes related to 3D models. The current version of the ontology contains almost 74000 classes and relations as well as 11 rules stored in a deductive RDF triple-store using a Sesame server, and that can be queried with a remote-access facility via a web server [30]. The ontology can be easily updated, just by entering or deleting triples and/or by modifying the set of rules, without having to change the reasoning algorithmic machinery used for answering queries. It is the strength of a declarative approach that allows a fine-grained domain-specific modeling and the exploitation of the result by a generic (domain-independent) reasoning algorithm.

MyCF features three distinct taxonomies linked by relations and rules: 

• Anatomical entities, such as *knee*, *shoulder*, and *hand*, denote parts of the human body, and give a formal description of canonical anatomy;

• Functional entities, such as *gait*, *breath*, and *stability*, denote the functions of the human body, and are the fundamental knowledge to explain the role of each anatomical entity;

• Finally, 3D scenes with entities such as *3D-object*, *3D-scene* define the content required to get 3D views of patient-specific anatomical entities described by 3D graphical models related to anatomical entities.

**Figure 1:**
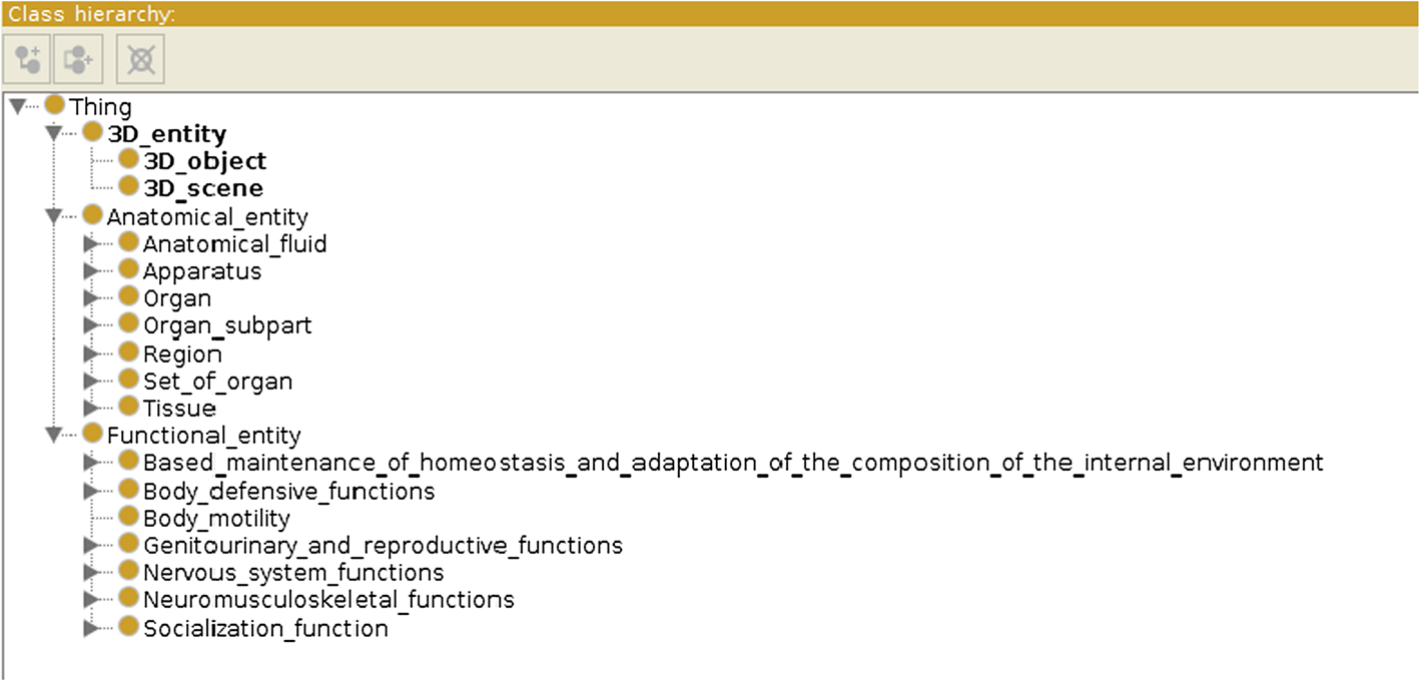
**Protégé display of the top classes of the three MyCF taxonomies.** The three top classes of MyCF are ‘3D entity’, ‘Anatomical entity’ (same as FMA) and ‘Functional entity’.

Figure 1 shows the top classes of the three taxonomies as they are displayed by the Protégé editor. We now in turn describe each of the taxonomies (of *anatomical entities*, *anatomical functions*, and *3D objects* respectively), the *relations* existing within and between them and the *inference rules* on which reasoning is performed.

### The taxonomy of anatomical entities

The taxonomy of anatomical entities of MyCF contains 69000 classes at the moment. It is inherited from the Foundational Model of Anatomy (FMA) ontology, in the sense that we have extracted from FMA a lot of terms that we have incorporated into MyCF. The correspondences between terms of the two ontologies are defined by means of the *owl:sameAs* relation. For example, to say that *mcf:Femur* corresponds to *fma:Femur*, we use the triple 〈*mcf:Femur*, *owl:sameAs*, *fma:Femur* 〉. However, we have skipped the top levels of the FMA taxonomy, that we have judged too general for our needs. For example, we skipped the FMA classes *Physical anatomical entity* and *Non-physical anatomical entity* declared in FMA as subclasses of the top FMA class *Anatomical entity*. We also skipped the two FMA classes *Material anatomical entity* and *Immaterial anatomical entity* appearing in FMA as subclasses of *Physical anatomical entity*, and the two FMA classes *Postnatal anatomical structure* and *Developmental anatomical structure* appearing as subclasses of the FMA subclass *Anatomical structure* of *Material anatomical entity*.

**Figure 2:**
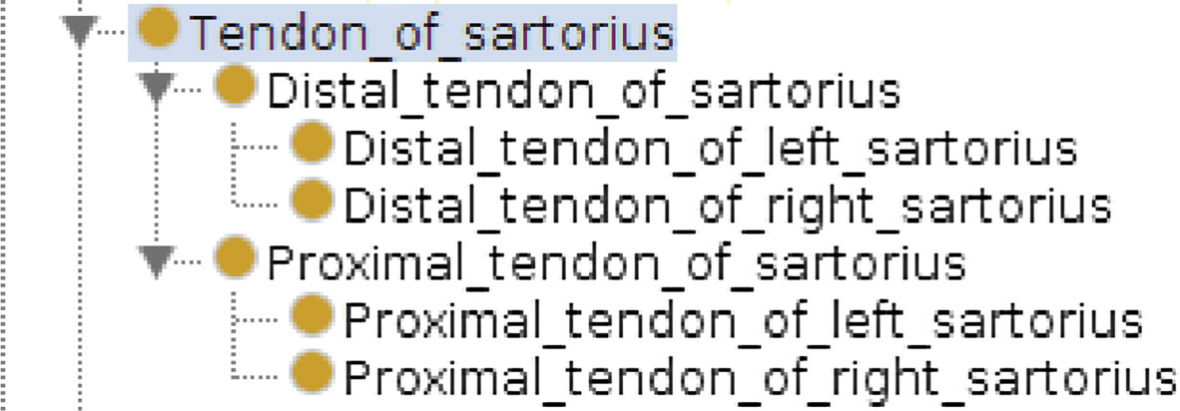
**Description of musculature in MyCF anatomical taxonomy (an extract about tendons of sartorius).** Muscles in MyCF are subdivided into different parts. A particular focused has been made on tendons that play important roles in biomedical simulations.

On the other hand, we have added to MyCF anatomical classes that are not present in FMA but relevant for biomechanical simulations and for 3D visualization such as tendons and other anatomical entities, especially for free limbs. Figure 2 illustrates the level of detail with which a musculature is described in MyCF.

**Figure 3:**
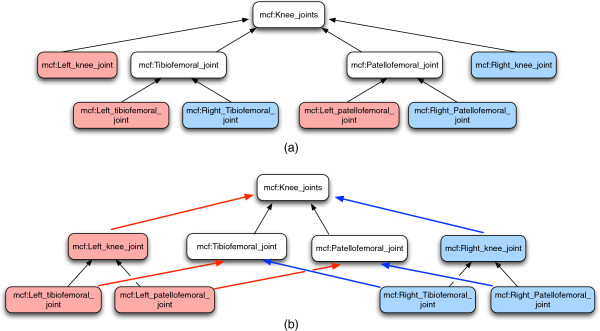
**Example of left and right structures of the knee joint.** Left-right structure of the *knee-joint* subclasses. **(a)** FMA taxonomy and **(b)** MyCF taxonomy. The novel properties *mcf:LeftSubClassOf* and *mcf:RightSubClassOf* are drawn in red and blue, respectively.

We have also introduced the possibility of making explicit the *left* and *right* specializations of anatomical entities. Many anatomical entities indeed can be *specialized* into two symmetric anatomical entities, representing its left and right version. For instance, the entity *knee* is specialized into the *left knee* and the *right knee*. Distinguishing the right and left versions of a given anatomical structure may be very important, for instance when linking anatomy with 3D models for simulation purposes. This provides new and convenient means of interacting with a 3D scene containing graphical entities that can be easily accessed, e.g. displaying the left knee. Our solution to this specialization in a semi-automatic and systematic way is the following one: 

• we add two new relations *mcf:leftSubClassOf* and *mcf:rightSubClassOf*, that we declare as specializations of the *rdfs:subClassOf* relation between anatomical entities using rules that will be detailed at the section ‘The taxonomy of anatomical functions’. These relations are not topological, in the sense that they do not aim at describing the absolute or relative displacement of an anatomical entity with respect to another, like the ones proposed in [8]. They aim at capturing the specialization of an anatomical concept, like the knee, in its left and right declensions;

• for every anatomical entity for which we want to declare its left and right specialization, e.g. *knee_joint*, we introduce two new names of classes, e.g. *Left_knee_joint* and *Right_knee_joint*, that we declare respectively as *mcf:leftSubClassOf* and *mcf:rightSubClassOf* of the anatomical entity. This is done by adding two RDF triples. For instance, in the case of the knee joint we have:

• 〈 Left_knee_joint mcf:leftSubClassOf knee_joint 〉,

• 〈 Right_knee_joint mcf:rightSubClassOf knee_joint 〉;

• we *iteratively replicate* all links, expressed as RDF triples, between an anatomical entity and one of its subclasses through the left and right specializations. For instance, in Figure 3, we report the result of these operations for the subtree of the anatomical taxonomy rooted in *knee_joint*.

Classifying entities with these new properties brings the following advantages: by querying on *SubClassOf* property, one can navigate the anatomical entities regardless of left/right parts, thus obtaining a more succinct representation of the data; by querying on *leftSubClassOf* and *rightSubClassOf* properties, one can navigate the complete taxonomy of the left and right specializations of an anatomical entity, if it is needed. Finally, we can easily relate a 3D object to the corresponding left or right specializations of anatomical entities, which is efficient to provide new means of selecting anatomical entities using the left and right concepts that are relevant in many 3D applications and biomechanical simulations.

In addition to the generic *rdfs:subClassOf* relation and its subproperties mcf:rightSubClassOf and mcf:rightSubClassOf that are the basis of the tree structure of the anatomical taxonomy, we have introduced the *mcf:PartOf* and *mcf:InsertOn* domain-specific relations. Then, we have declared in the form of RDF triples and rules that will be explained as knowledge on how anatomical entities are related by these two properties: 

• The property *mcf:PartOf* is used to make explicit the subparts of anatomical entities, which is an important anatomical knowledge. For example, a *joint* is a *part of* the *articular system* (but joint is *not* a subclass of an articular system), is declared by adding to the ontology data base the RDF triple:

• 〈 mcf:Joint mcf:PartOf mcf:Articular_System 〉.

• Note that, like for FME (the explorer of FMA), the *mcf:PartOf* relation can be chosen for defining the tree structure through which the user wants to visualize the anatomical entities in 3D, as an alternative to the tree structure defined by the *rdfs:subClassOf* relation.

• The property *mcf:InsertOn* is used to specify attach points of anatomical entities. This knowledge is important in anatomy and also for biomechanical simulation purposes. For instance, the *distal tendon of right sartorius* is *inserted on* the *Medial part of proximal epiphysis of right tibia*, is expressed by adding the RDF triple:

• 〈 mcf:Distal_Tendon_Of_Right_Sartorius mcf:InsertOn mcf:Medial_part_of_proximal_epiphysis_of_right_tibia 〉.

In the current version of MyCF, there are 4000 RDF triples involving the property *mcf:PartOf*, and 850 RDF triples involving the property *mcf:InsertOn*.

### The taxonomy of anatomical functions

The taxonomy of anatomical functions of MyCF is the true added-value of MyCF that distinguishes it from the state-of-the-art anatomical ontologies. It contains 4000 classes at the moment, most of the terms used to denote them come from the ICF terminology. The anatomical functions are structured using two relations: 

• the generic *rdfs:subClassOf* relation between functions. For instance, the *extension of the knee* is a subclass of the *simple movement* function, is expressed by the RDF triple:

• 〈 mcf:Extension_Of_The_Knee rdfs:subClassOf mcf:Simple_Movement 〉

• and the domain-specific relation *mcf:IsInvolvedIn* which plays a role analogous to that of the *partOf* relation between anatomical entities. For example, the *eversion of the foot is involved in* the *mobility of ankle joints*, is expressed by adding the RDF triple:

• 〈 mcf:Eversion_Of_The_Foot mcf:IsInvolvedIn mcf:Mobility_Of_Ankle_Joints 〉

• Notice that the *eversion of the foot* is not a *subclass of* the *mobility of ankle joints*.

In the current version of MyCF, there are 4000 RDF triples specifying *rdfs:subClassOf* relations between functions, and 1300 RDF specifying *mcf:IsInvolvedIn* relations between functions.

The real added-value of MyCF is to link the taxonomy of functions with the anatomical taxonomy to make explicit the functional roles of anatomical entities. Exploiting the relationships between anatomical and functional entities is decisive to retrieve the entities participating to some functions, and vice-versa. In particular, this is crucial for medical diagnosis and it is also of key importance to be able to display/select interactively 3D geometric entities. To address this issue, we have introduced two domain-specific relations, *mcf:hasFunction* and *mcf:contributesTo* to describe how an anatomical entity contributes to a given function. The former is used to denote that an anatomical entity, as a whole, realizes a given function. The latter is used to denote that an anatomical entity simply contributes to the realization of a given function, but taken alone it may not be sufficient to execute this function. 

• The relation *mcf:hasFunction* relates an anatomical entity with the function(s) that it realizes. For instance, we can declare that the function *knee movement* is performed by the *knee* by the following RDF triple:

• 〈 mcf:Knee mcf:hasFunction mcf:Knee_Movement 〉

• Similarly, we can make explicit the functions of *ensuring sliding motion of articular surface* and *ensuring transmission and amortization of charges* of *joint cartilages* by the two following RDF triples:

• 〈 mcf:Joint_Cartilage mcf:hasFunction mcf:Ensure_Sliding_Motion_Of_Articular_Surface 〉

• 〈 mcf:Joint_Cartilage mcf:hasFunction mcf:Ensure_Transmission_And_Amortization_Of_Charges 〉

• The relation *mcf:contributesTo* is a weaker relation than the relation *mcf:hasFunction*, which allows to specify that a given anatomical entity contributes to the realization of a given (set of) function(s). For instance, despite the fact that the *toe* does not *have as function* the *body stability*, the *toe contributes to* the *body stability*. This can be expressed by the RDF triple:

• 〈 mcf:Toe mcf:contributesTo mcf:Body_Stability 〉

• Note that, as it will be explained in the next section, this triple can be declared or inferred by rules. We will also show how we express by a rule that the relation *mcf:hasFunction* is stronger than the relation *mcf:contributesTo*, which enables to infer that any anatomical enity that is declared as having as function a given anatomical function, contributes to this function a fortiori.

In the current version of MyCF, there are 700 RDF triples specifying *mcf:hasFunction* between anatomical entities and anatomical functions, and 500 RDF specifying a *mcf:contributesTo* relation between anatomical entities and anatomical functions.

### The taxonomy of 3D objects

**Figure 4:**
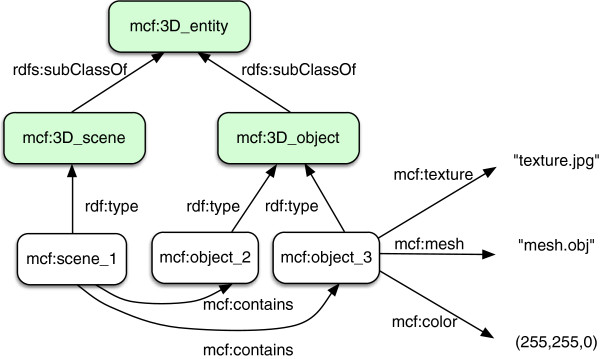
**3D taxonomy in MyCF.** 3D taxonomy of MyCF is basic with only three classes. The individual, for instance, called object_3 is an mcf:3D_object that has a geometry (obj file) and a texture (jpg file) allowing a 3D visualization and interaction.

The taxonomy of 3D objects of MyCF is simple but mandatory to connect the anatomical entities and their functions to graphic entities used to interact with these 3D objects. It also illustrates well the declarative way to connect additional knowledge for different purposes to a given ontology. Here, we want to connect to the anatomical ontology (patient-specific) to 3D geometric models displaying a body part, so that the different 3D objects contained in the scene are related to the anatomical entities they describe, thus providing the user with new means to select/display these entities using the knowledge embedded in the taxonomies of anatomical entities and anatomical functions as well as their relationships. This is a new scheme to avoid the selection/display of these entities using purely geometry-based approaches. The proposed taxonomy aims at defining the smallest content enabling elementary tasks to display/select 3D objects though this can be enriched to refer to geometric criteria for these tasks that would add other entities in this taxonomy. Our approach for doing so consists in: 

• Designing a taxonomy of geometric objects (shown in Figure 4) made of two classes respectively called *mcf:3D-scene* and *mcf:3D-object*, a relation called *mcf:Contains* having the class *mcf:3D-model* as domain and the class *mcf:3D-object* as range, and four relations respectively called *mcf:Position*, *mcf:hasMesh*, *mcf:hasTexture* and *mcf:hasColour* respectively, in order to possibly relate each specific 3D-object to a position matrix, a mesh file, a texture file, and a color,

• linking this 3D geometry taxonomy to the anatomical taxonomy through two relations called respectively *mcf:Describes* relating instances of the class *mcf:3D-object* to instances of the class *fma:Anatomical Entity*, and *mcf:Displays* relating instances of the class *mcf:3D-scene* to instances of *fma:Anatomical Entity* or of *mcf:Anatomical Function*.

• declaring each new patient-specific 3D model that we acquire as an *instance* of the class *3D-scene*, by a RDF triple: 〈 mcf:id rdf:type 3D-scene 〉 where mcf:id denotes an identifier (e.g., an URI) where the file modeling the 3D-scene is stored, and stating which body part or function it displays by an RDF triple, for instance:

• 〈 mcf:id mcf:Displays mcf:Knee 〉

• identifying all the 3D-objects segmented within the 3D-scene and corresponding to anatomical entities as instances of the class *3D-object*, for which a number of RDF triples are declared to specify that they identify 3D-objects *contained* in the 3D-scene from which they have been extracted, and that they *describe* the corresponding anatomical entity.

**Figure 5:**
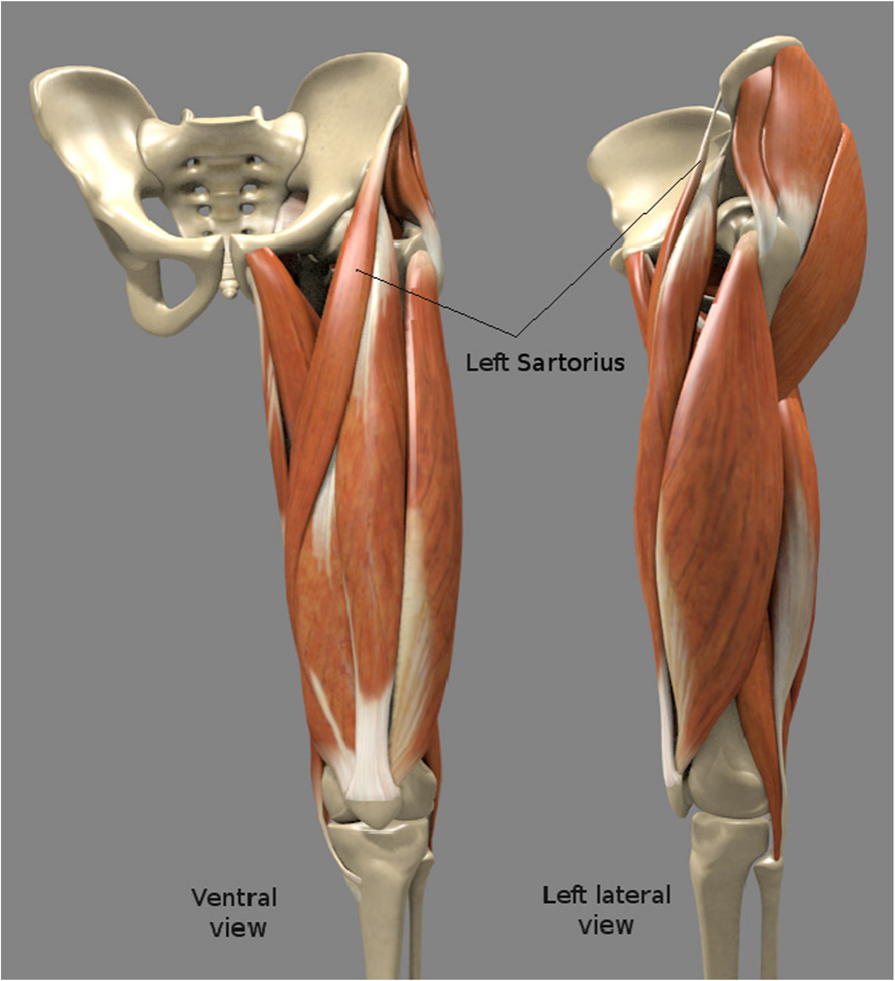
**Example of a 3D scene containing complex 3D anatomical models.** 3D-model of the proximal part of the left lower limb. Only the left sartorius is pointed out.

For instance, the 3D-scene displayed in Figure 5, stored in a file identified by mcf:id, in which the different coloured 3D-objects corresponding to muscles and bones have been extracted by segmentation, would be described in myCF by the following RDF triples:

〈 mcf:id rdf:type mcf:3D-scene 〉 〈 mcf:id mcf:Displays mcf:Leg 〉

〈 mcf:id mcf:Contains mcf:id1 〉 〈 mcf:id1 rdf:type mcf:3D-object 〉

〈 mcf:id mcf:Contains mcf:id2 〉 〈 mcf:id2 rdf:type mcf:3D-object 〉

〈 mcf:id mcf:Contains mcf:id3 〉 〈 mcf:id3 rdf:type mcf:3D-object 〉

....

〈 mcf:id1 mcf:Describes mcf:Left_sartorius 〉

〈 mcf:id1 mcf:hasMesh ¨..\geometries\l_sartorius.obj ¨ 〉

〈 mcf:id2 mcf:Describes mcf:Left_bicepsfemoris 〉

〈 mcf:id2 mcf:hasMesh ¨..\geometries\l_bicepsfemoris.obj ¨ 〉

〈 mcf:id3 mcf:Describes mcf:Left_semimembranosus 〉

〈mcf:id2 mcf:hasMesh ¨..\geometries\l_semimembranosus.obj ¨ 〉

.....

**Figure 6:**
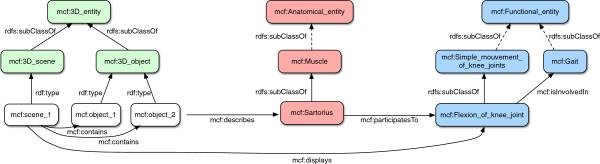
**The general structure of MyCF ontology (extract).** The three taxonomies of MyCF are interconnected allowing a high level of knowledge expression.

Figure 6 summarizes the structure of MyCF ontology made of its three taxonomies interrelated by relations.

### The inference rules

The inference rules of MyCF express complex connections between relations. They allow the ontology designer to declare part of his/her domain knowledge in the form of abstract rules. These rules capture in a very compact way implicit facts that can be made explicit on demand or at query time by an inference mechanism. This mechanism is automatic and consists in applying the rules on the explicit facts declared and stored as RDF triples, in all the possible manners satisfying the conditions of these rules. For each possible instantiation of the variables (denoted by a name starting by ?) appearing in the condition part of a given rule such that all its conditions are satisfied by explicit facts, the new facts corresponding to the (appropriately instantiated) conclusion of the rule are added. This saturation process is iterated as long as new facts can be produced. The termination is guaranteed by the form of the rules that are considered. They correspond to *safe* rules, also called Datalog rules: all the variables appearing in the conclusion of a rule also appears in the condition part. This contrasts with description logics axioms or with ${\text{Datalog}}_{-}^{+}$ rules [31] in which we can infer that there exists (unknown) individuals verifying a given property.

The rules that are considered in the current version of MyCF are the following ones. It is important to note however that adding, removing or modifying a rule is very simple and does not impact the inference mechanism that remains unchanged as long as the rules added that are safe ones.

The three following rules express the transitivity of the generic relation rdfs:subClassOf, as well as of the domain-specific relations mcf:PartOf between anatomical entities and mcf:IsInvolvedIn between anatomical functions, respectively.

(R1) $$\begin{array}{lll} & \;\text{IF}\langle \;\text{?a rdfs:subClassOf ?c}\;\rangle \text{AND}\langle \;\text{?c rdfs:subClassOf ?b}\;\rangle \; & \\ & \;\text{THEN}\langle \;\text{?a rdfs:subClassOf ?b}\;\rangle \; \end{array}$$

(R2) $$\begin{array}{lll} & \;\text{IF}\langle \;\text{?a mcf:PartOf ?c}\;\rangle \text{AND}\langle \;\text{?c mcf:PartOf ?b}\;\rangle \; & \\ & \;\text{THEN}\langle \;\text{?a mcf:PartOf ?b}\;\rangle \; \end{array}$$

(R3) $$\begin{array}{lll} & \;\text{IF}\langle \;\text{?a mcf:IsInvolvedIn ?c}\;\rangle \text{AND}\langle \;\text{?c mcf:IsInvolvedIn ?b}\;\rangle \; & \\ & \;\text{THEN}\langle \;\text{?a mcf:IsInvolvedIn ?b}\;\rangle \; \end{array}$$

The three following rules express specializations of relations: mcf:LeftSubClassOf and mcf:RightSubClassOf are both two specializations of rdfs:subClassOf ; and the relation mcf:hasFunction (between an anatomical entity and an anatomical function) is more specific (i.e., more precise) than the relation mcf:contributesTo (between an anatomical entity and an anatomical function).

(R4) $$\begin{array}{ll} & \;\text{IF}\langle \;\text{?a mcf:LeftSubClassOf ?b}\;\rangle \text{THEN}\langle \;\text{?a rdfs:subClassOf ?b}\;\rangle \; \end{array}$$

(R5) $$\begin{array}{ll} & \;\text{IF}\langle \;\text{?a mcf:RightSubClassOf ?b}\;\rangle \text{THEN}\langle \;\text{?a rdfs:subClassOf ?b}\;\rangle \; \end{array}$$

(R6) $$\begin{array}{ll} & \;\text{IF}\langle \;\text{?a mcf:hasFunction ?b}\;\rangle \text{THEN}\langle \;\text{?a mcf:contributesTo ?b}\;\rangle \; \end{array}$$

Finally, the following rules express connections that hold in the domain of anatomy between the relations rdfs:subClassOf and mcf:InsertOn, rdfs:subClassOf and mcf:IsInvolvedIn, rdfs:subClassOf and mcf:contributesTo, mcf:contributesTo and mcf:IsInvolvedIn, mcf:PartOf and mcf:InsertOn respectively.

For example, the first rule says that if a given class representing an anatomical entity ?a (e.g., Sartorius) is a subclass of an anatomical entity ?c (e.g., Muscle) that is known to be inserted on an anatomical entity ?b (e.g., Bone), then ?a is inserted on ?b (Sartorius inserts on a Bone).

(R7) $$\begin{array}{lll} & \;\text{IF}\langle \;\text{?a rdfs:subClassOf ?c}\;\rangle \text{AND}\langle \;\text{?c mcf:InsertOn ?b}\;\rangle \; & \\ & \;\text{THEN}\langle \;\text{?a mcf:InsertOn ?b}\;\rangle \; \end{array}$$

(R8) $$\begin{array}{lll} & \;\text{IF}\langle \;\text{?a mcf:IsInvolvedIn ?c}\;\rangle \text{AND}\langle \;\text{?c rdfs:subClassOf ?b}\;\rangle \; & \\ & \;\text{THEN}\langle \;\text{?a mcf:IsInvolvedIn ?b}\;\rangle \; \end{array}$$

(R9) $$\begin{array}{lll} & \;\text{IF}\langle \;\text{?a mcf:contributesTo ?c}\;\rangle \text{AND}\langle \;\text{?c rdfs:subClassOf ?b}\;\rangle \; & \\ & \;\text{THEN}\langle \;\text{?a mcf:contributesTo ?b}\;\rangle \; \end{array}$$

(R10) $$\begin{array}{lll} & \;\text{IF}\langle \;\text{?a mcf:contributesTo ?c}\;\rangle \text{AND}\langle \;\text{?c mcf:IsInvolvedIn ?b}\;\rangle \; & \\ & \;\text{THEN}\langle \;\text{?a mcf:contributesTo ?b}\;\rangle \; \end{array}$$

(R11) $$\begin{array}{lll} & \;\text{IF}\langle \;\text{?a mcf:InsertOn ?c}\;\rangle \text{AND}\langle \;\text{?c mcf:PartOf ?b}\;\rangle \; & \\ & \;\text{THEN}\langle \;\text{?a mcf:InsertOn ?b}\;\rangle \; \end{array}$$

The point is that we can easily add rules crossing the anatomy domain and the 3D domain, to express, for instance, conventional colors associated with the visualization of some organs (such as bones, muscles, and so on). The following rule expresses that the conventional color for visualizing bones in anatomy is yellow:

(R12) $$\begin{array}{lll} & \text{IF}\langle \;\text{?x rdf:type mcf:3D-object}\;\rangle \text{AND}\langle \;\text{?x mcf:Describes ?y}\;\rangle \; & \\ & \text{AND}\langle \;\text{?y rdfs:subClassOf mcf:Bone}\;\rangle \; & \\ & \text{THEN}\langle \;\text{?x mcf:hasColour ‘yellow’}\;\rangle \; \end{array}$$

### Querying: illustration by example

**Figure 7:**
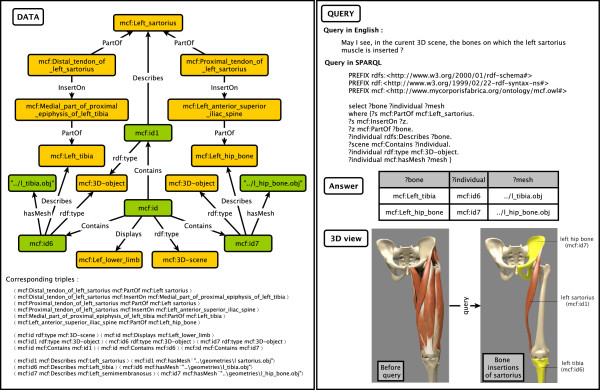
**Example of querying about anatomy of the sartorius.** The graph on the left is a visual representation of data. The query about left sartorius, translated in SPARQL, gives the bones on which the tendons of sartorius are inserted. In the final 3D scene the sartorius is showed alone and the corresponding bones are highlighted in yellow.

In the Figure 7, we illustrate a complete example from query to 3D visualization. Data are presented as a graph with corresponding RDF triples on the bottom. The query is explained in English and translated in SPARQL. The answers are used to select and highlight corresponding 3D models in the 3D scene.

## Conclusions

We have described MyCF with a particular emphasis on its ontology structure, showing how the FMA ontology can be used as basis of the anatomical description of human bodies and empowered with a taxonomy of anatomical functions conforming to the ICF terminology. We have introduced new concepts that are particularly useful for checking the anatomical validity of 3D models containing multiple anatomical entities, also for selecting sets of anatomical entities on the basis of functions rather than being bound to geometric approaches that are not efficient enough to process complex 3D geometric configurations.

These high level functionalities can be achieved thanks to the combination of different types of knowledge related to anatomy. The reasoning capabilities brought by the inference rules increase the power of realizing complex tasks by reducing them to querying a knowledge base implemented as a deductive database.

The main interest of this approach is its declarativity that makes possible for domain experts to enrich the knowledge base at any moment through simple editors without having to change the algorithmic machinery.

This provides MyCF software environment a flexibility to process and add semantics on purpose for various applications that incorporate not only symbolic information but also 3D geometric models representing anatomical entities as well as other symbolic information like the anatomical functions.

The MyCF ontology is at the heart of the the MyCF Browser: a tool for exploring anatomical 3D models [32].

Further work will address the use of this environment to feed a bio-simulation engine with the appropriate anatomical entities so that mechanical simulations can be easily set up and extract the required geometric information from the 3D models of anatomical entities.

## Methods

Through the presentation of MyCF, we develop a novel and promising vision of ontologies equipped with inference algorithms, that enables a declarative assembly of different models to obtain composed models guaranteed to be anatomically valid while capturing the complexity of human anatomy.

### Methodology overview of the design of MyCF ontology

We have designed a unifying representation framework to combine several types of structured knowledge about anatomy.

For the types of anatomical knowledge for which ontologies or terminologies exist, our approach is to enrich them while remaining conform to them. The descriptions in MyCF’s ontology of the anatomical concepts and the human body functions are thus conform respectively to the Foundational Model of Anatomy (FMA) [2] and to the International Classification of Functioning, Disability and Health (ICF) [11]. In fact, MyCF’s ontology both enriches and links together two standard taxonomies that have been developed separately and independently.

For incorporating 3D models in MyCF ontology, in order to follow a unifying approach, we have chosen to define a taxonomy of 3D scenes and 3D objects, and to relate it to the taxonomies of anatomical entities and functions through relations.

One particularity of MyCF is to use (generic and specific) relations both to structure each taxonomy but also to establish bridges between them. Inference rules are used to express how relations interact.

We give now some details on our methodological choices both for incorporating 3D models and inference rules.

#### ***3D models***

We want to be able to incorporate different types of 3D models. Some 3D models can describe patient-specific body parts acquired by CT (Computerized Tomography) or MRI (Magnetic Resonance imaging) scans. In this case, the 3D models are obtained by reconstruction using classic surface modeling techniques. The 3D models used to illustrate this article are based on the Zygote human anatomy collections [33]. The resulting 3D models are mesh-based files associated with position matrices and texture files for 3D view rendering. The storage and the processing of the files describing the 3D objects are specially time and memory consuming. Our approach is to disconnect the identification of these files from their storage and processing, and to connect them to the ontology through their identifiers: each file’s identifier is declared as an instance of a 3D scene capturing an anatomical structure, a body part, or a human body function in the ontology. By segmentation, the 3D scene is decomposed into components that are in turn declared as instances of 3D objects describing the anatomical entities declared in the ontology as parts of the given anatomical structure.

#### Inference rules

We have chosen the formalism of rules to express properties of relations (such as transitivity) but also properties or constraints between domain-specific relations. For instance, the following rule involving two domain-specific relations (ContributesTo and IsInvolvedIn) expresses that any anatomical entity ?*C* participating to a function ?*F* that is involved in a function ?*F*^′^ contributes to this function ?*F*^′^ too:

$$\begin{array}{lll} & \text{IF}\langle \;\text{?C mcf:ContributesTo ?F}\;\rangle \text{AND}\langle \;\text{?F mcf:IsInvolvedIn ?F’}\;\rangle \; & \\ & \text{THEN}\langle \;\text{?C mcf:ContributesTo ?F’}\;\rangle \; & \end{array}$$

Such a rule is a compact formula that enables to infer as many instantiated facts as there exist pairs of facts satisfying its conditions. For example, using this rule, we can infer that the muscle **sartorius** (but also the **biceps femorus** muscle) contributes to the function of **movement of knee** from the facts that **sartorius** (but also the **biceps femorus**) contributes to the function **knee flexion** and that the function **knee flexion** is involved in the function **movement of knee**. Similarly, by using the same rule, we can infer that the different muscles (such as **Tensor fascia lata**, **Rectus femoris**, **Vastus lateralis**, **Vastus medialis**, and **Vastus intermedius**) contributing to the function **knee extension** contribute too to the **movement of knee** since **knee extension** is involved in the **movement of knee**. This is a simple but powerful piece of knowledge that can guide diagnosis by iteratively identifying the anatomical entities to check in case of dysfunction of the movement of a knee. It can also help setting up an appropriate 3D scene or a biomechanical simulation. In both cases, the interest is to select the anatomical entities relevant to display them and meet the user’s needs or to select the anatomical entities to simulate a knee movement. Rules can be very useful too for guiding image segmentation or image registration in medical imaging. For instance, a rule stating that every sinovial joint has an articular capsule can guide automatic segmentation of patient-specific images.

### Semantic technologies used for building and exploiting MyCF

In order to make it easy to connect MyCF to the Linked Data cloud [34], we have followed the recommendations of W3C and we have chosen the RDF(S) language for expressing MyCF ontology.

RDF [35] is a standard notation recommended by the W3C for the semantic Web composed of Web data and (simple) ontologies. RDF (Resource Description Framework) provides a simple language for describing annotations about Web resources identified by URIs. An RDF fact consists of a triple made of a subject, a predicate and an object. It expresses a relationship denoted by the predicate between the subject and the object. In a triple, the subject, but also the predicate, are URIs pointing to Web resources, whereas the object may be either a URI or a literal representing a value. RDFS is the schema language for RDF. It allows specifying a number of useful constraints on the individuals and relationships used in RDF triples. In particular, it allows declaring objects and subjects as instances of certain classes. In addition, inclusion statements between classes and properties make it possible to express semantic relations between classes and between properties. Finally, it is also possible to semantically relate the domain and the range of a property to some classes. The point is that these constraints can be written in triple notation, i.e., RDFS statements can be written using RDF as a notation. Therefore, a RDF data store can contain in the same format triples expressing that a given acquisition file (identified by a given URL u) is an instance of an anatomical structure (for instance the patella), and triples describing knowledge known in the domain of anatomy about this structure (for instance that the patella is a circular-triangular bone, and that it is part of the knee):

〈 u rdf:type mcf:Patella 〉 〈 mcf:Patella rdfs:subClassOf mcf:CircularTriangularBone 〉 〈 mcf:Patella mcf:PartOf mcf:Knee 〉

As ontology editors, we have used Protégé [36] and TopBraid Composer [37]. Protégé is supported by a strong community of developers and academic, government and corporate users. The Protégé open source platform supports modeling ontologies in a variety of formats via a web client or a desktop client. TopBraid is a commercial tool specifically designed for RDF, which is also available as free version.

**Figure 8:**
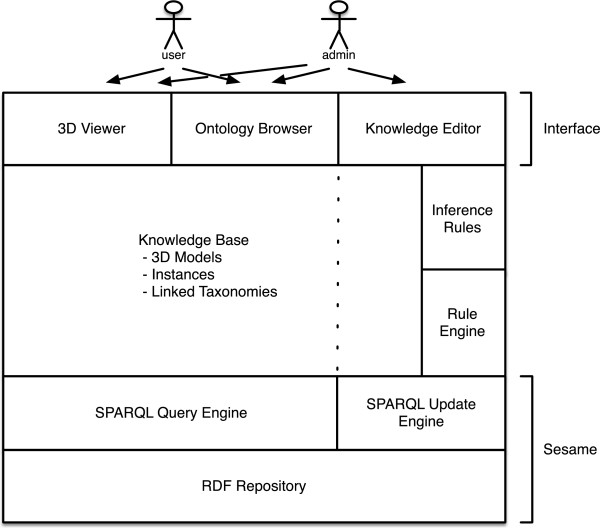
**Architecture of the MyCF environment.** Overview of the architecture of MyCF.

Finally, we have chosen to store and process the resulting ontology as a deductive RDF triple-store using a Sesame server. Sesame [38] is a de-facto standard framework for processing RDF data. This includes parsing, storing, inferencing and querying of/over such data. It offers an easy-to-use API that can be connected to all leading RDF storage solutions. Sesame fully supports the SPARQL [39] query language for expressive querying and offers transparent access to remote RDF repositories using the exact same API as for local access. However Sesame currently has no built-in support for custom inference rules. Therefore, we had to implement a rule engine on top of it in order to enable sound and complete deductive capabilities. This architecture is of course modular and adjustable. For instance, it is possible to change the triple-store server, or to use an external reasoner supporting Datalog rules for saturating the data. Figure 8 sketches the general architecture of the MyCF environment.

## Competing interests

The authors declare that they have no competing interests.

## Authors’ contributions

OP conceived the original idea, contributed to the models (3D models and ontologies), participated to the software development of a preliminary version of My Corporis Fabrica, and supervised this project. FU contributed to the ontological modeling and to the design of the overall architecture, implemented the rule engine, and is the main software developer. VF is the main developer of the taxonomy of anatomical functions. JCL contributed to the models (3D models and ontologies). MCR contributed to the ontological modeling and to the design of the overall architecture, and co-supervised this project. All authors read and approved the final manuscript.
